# Supplementary figures and images for: Fabrication of calixarene-grafted bio-polymeric magnetic composites for magnetic solid phase extraction of non-steroidal anti-inflammatory drugs in water samples
Source: PeerJ. 2018 Jul 6;6:e5108. doi: 10.7717/peerj.5108 (PMC6037154; doi:10.7717/peerj.5108)

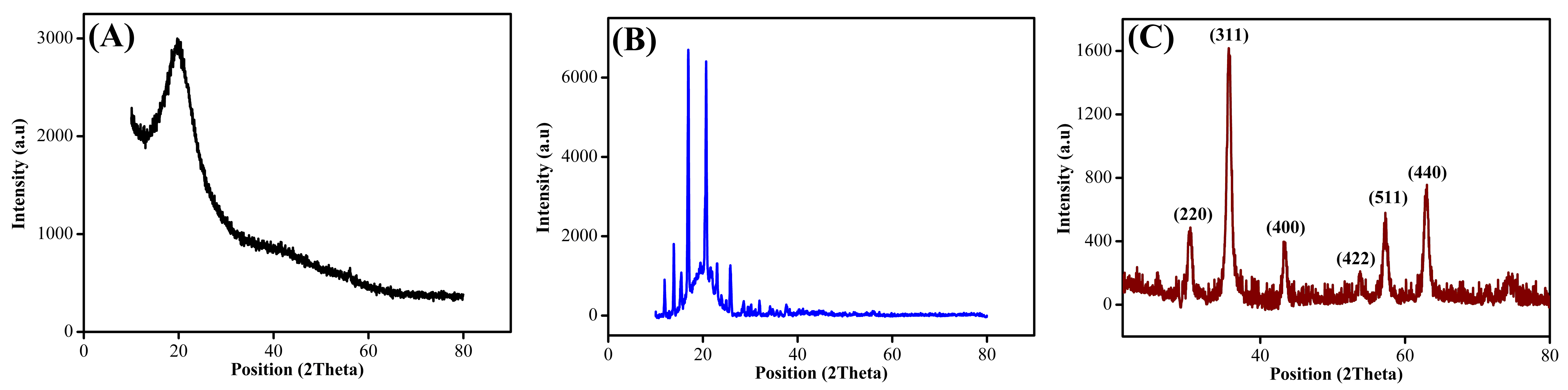

Supplement: Figure S1 — XRD pattern of (A) Sp-TDI, (B) Sp-TDI-calix and (C) MSp-TDI-calix. [file peerj-06-5108-s001.png]

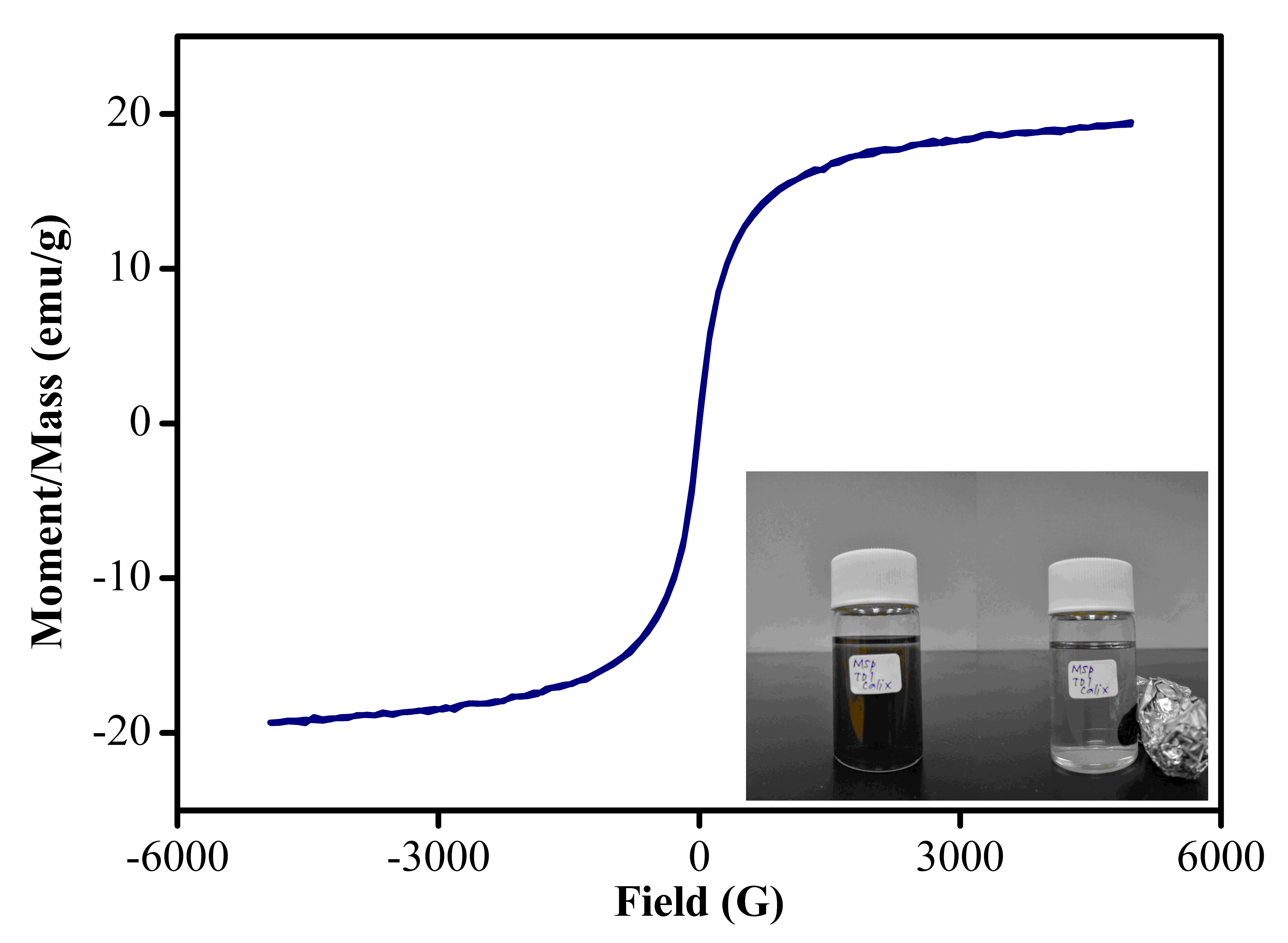

Supplement: Figure S2 — Magnetization curve of MSp-TDI-calix. The inset shows photograph of magnetic biopolymer adsorbent dispersed in aqueous solution (left) and separated from aqueous solution under an external magnetic field (right). [file peerj-06-5108-s002.png]

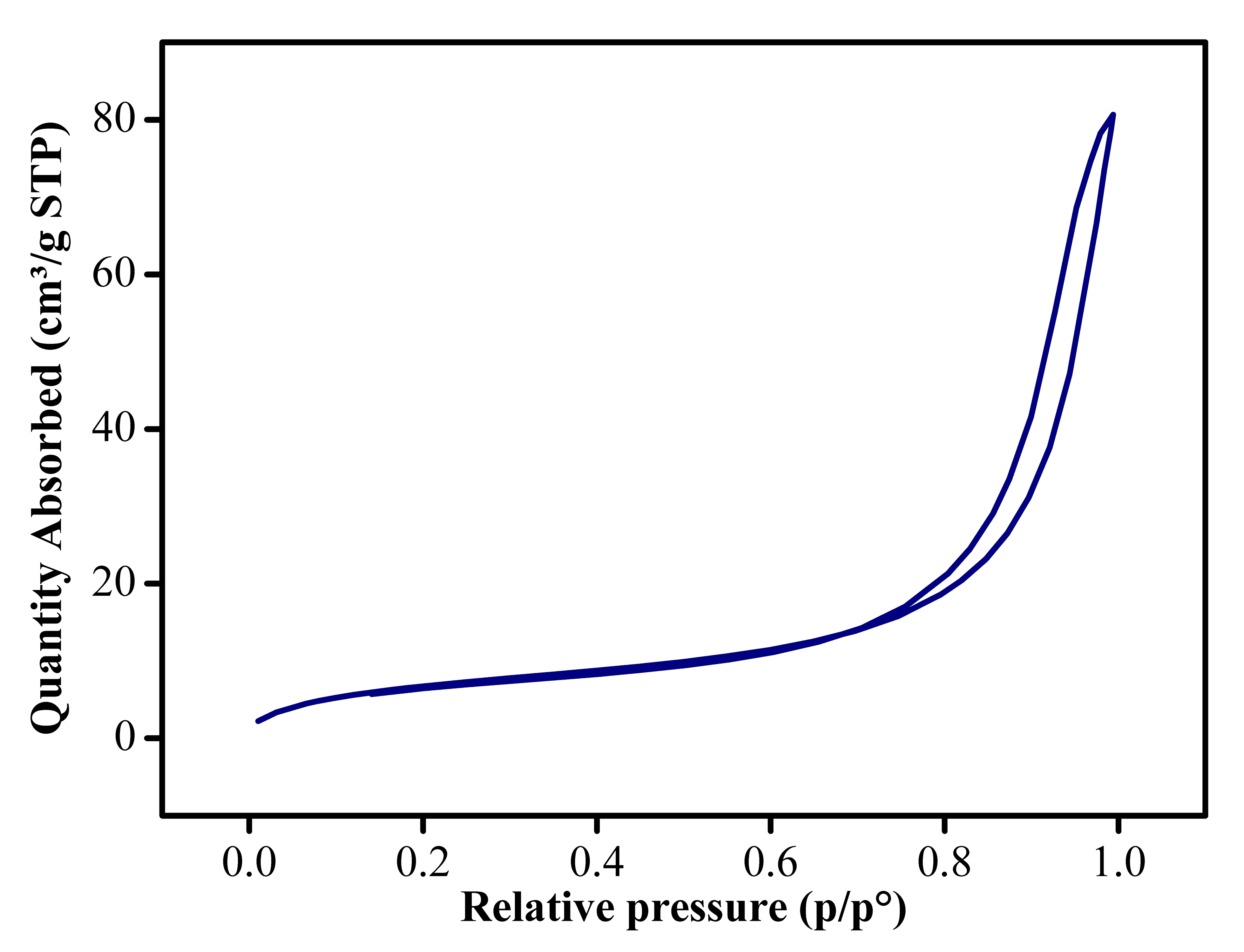

Supplement: Figure S3 [file peerj-06-5108-s003.png]

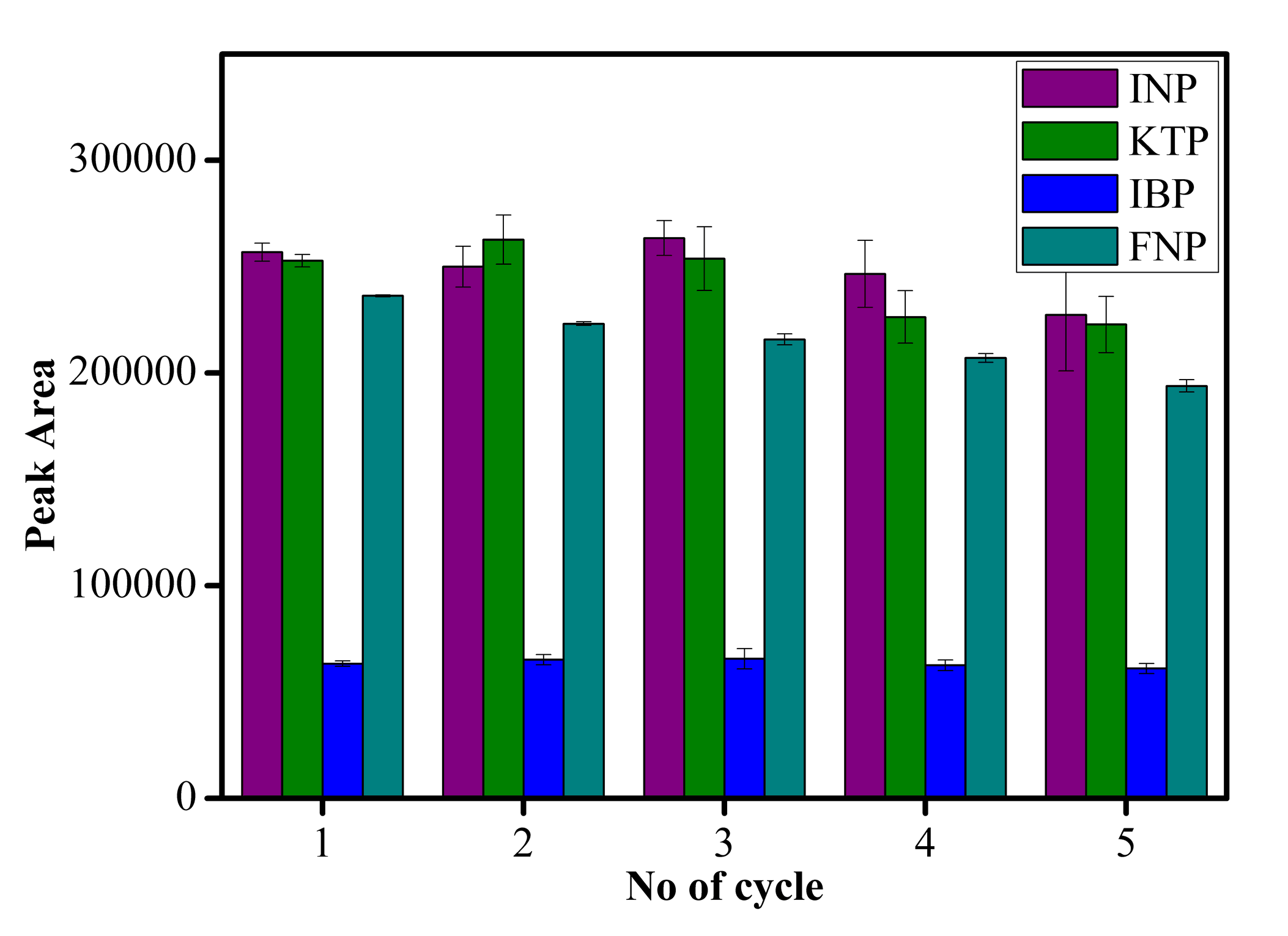

Supplement: Figure S4 — Reusability of the sorbent for the extraction of NSAIDs using MSp-TDI-calix and analysis using HPLC-DAD. HPLC conditions: acidified (1% with acetic acid) water/acetonitrile (50:50 v/v) as a mobile phase at a flow rate of 1 mL min −1, the HPLC column temperature was set at 40 °C, the sample injection volume was 10 µL, the DAD detection for the selected NSAIDs was carried out at multiple wavelengths i.e., 281, 255, 271 and 219 nm for INP, KTP, IBP and FNP respectively. [file peerj-06-5108-s004.png]
