# Supplementary material for: Fabrication of calixarene-grafted bio-polymeric magnetic composites for magnetic solid phase extraction of non-steroidal anti-inflammatory drugs in water samples
Source: PeerJ. 2018 Jul 6;6:e5108. doi: 10.7717/peerj.5108 (PMC6037154; doi:10.7717/peerj.5108)
Supplement: Table S1 — Physical properties of Sporopollenin, Sp-TDI-calix and MSp-TDI-calix. [file peerj-06-5108-s005.docx]

Table S1 Physical properties of Sporopollenin, Sp-TDI-calix and MSp-TDI-calix

| Sample | Surface area (m^2^/g) | Pore volume (cm^3^/g) | Pore size (nm) |
| --- | --- | --- | --- |
| Sporopollenin | 2.27 | 0.00147 | 2.60 |
| Sp-TDI-calix | 2.08 | 0.00169 | 3.26 |
| MSp-TDI-calix | 26.5 | 0.125 | 18.8 |
